# Prescription quantity and duration predict progression from acute to chronic opioid use in opioid-naïve Medicaid patients

**DOI:** 10.1371/journal.pdig.0000075

**Published:** 2022-08-25

**Authors:** Drake G. Johnson, Vy Thuy Ho, Jennifer M. Hah, Keith Humphreys, Ian Carroll, Catherine Curtin, Steven M. Asch, Tina Hernandez-Boussard

**Affiliations:** 1 Department of Medicine, Stanford University, Stanford California, United States of America; 2 Department of Surgery, Stanford University, Stanford California, United States of America; 3 Department of Anesthesiology, Perioperative, and Pain Medicine, Stanford University, Stanford California, United States of America; 4 Department of Psychiatry, Stanford University, Stanford California, United States of America; 5 Veterans Affairs Health Care System, Palo Alto, California United States of America; 6 Department of Biomedical Data Sciences, Stanford University, Stanford California, United States of America

## Abstract

Opiates used for acute pain are an established risk factor for chronic opioid use (COU). Patient characteristics contribute to progression from acute opioid use to COU, but most are not clinically modifiable. To develop and validate machine-learning algorithms that use claims data to predict progression from acute to COU in the Medicaid population, Adult opioid naïve Medicaid patients from 6 anonymized states who received an opioid prescription between 2015 and 2019 were included. Five machine learning (ML) Models were developed, and model performance assessed by area under the receiver operating characteristic curve (auROC), precision and recall. In the study, 29.9% (53820/180000) of patients transitioned from acute opioid use to COU. Initial opioid prescriptions in COU patients had increased morphine milligram equivalents (MME) (33.2 vs. 23.2), tablets per prescription (45.6 vs. 36.54), longer prescriptions (26.63 vs 24.69 days), and higher proportions of tramadol (16.06% vs. 13.44%) and long acting oxycodone (0.24% vs 0.04%) compared to non- COU patients. The top performing model was XGBoost that achieved average precision of 0.87 and auROC of 0.63 in testing and 0.55 and 0.69 in validation, respectively. Top-ranking prescription-related features in the model included quantity of tablets per prescription, prescription length, and emergency department claims. In this study, the Medicaid population, opioid prescriptions with increased tablet quantity and days supply predict increased risk of progression from acute to COU in opioid-naïve patients. Future research should evaluate the effects of modifying these risk factors on COU incidence.

## Author summary

Prescription opioids in the United States contribute to opioid-related overdose deaths. Evidence suggests the Medicaid population has a greater likelihood of opioid-related mortality. However, the current standard for postoperative pain management often doesn’t account for previous opioid use and/or misuse. Machine learning can help healthcare systems identify patients at risk of progression from opioid naïve to chronic opioid use (COU) in Medicaid patients. In this cohort study of 180,000 opioid naïve Medicaid patients, machine learning models accurately identified opioid naïve patients at risk of progression to COU. Features of the initial opioid prescription were strong predictors of progression to COU, including number of tablets, prescription length, and prescription of oxycodone. We found that prescription quantity and length are discrete features related to opioid prescribing that are predictive of COU incidence. This research suggests that stronger prescription guidelines are needed for opioid prescriptions among opioid naïve patients.

## Introduction

Prescription opioids in the United States contribute to opioid-related overdose deaths and an annual economic burden exceeding $78 billion [1,2]. State-level policy interventions to address the opioid crisis have included prescription drug monitoring programs, prior authorization requirements, and limits on prescription length and dosage [3–5]. Reported outcomes are heterogenous; recent evidence suggests state-level drug policies may reduce prescription opioid misuse in the Medicare population when participation in prescription drug monitoring programs is mandatory.[6] Finding modifiable factors that can positively impact opioid-related endpoints is needed to guide these policies.

Identifying features of responsible opioid prescribing is particularly important in Medicaid patients, because they are at increased risk of developing chronic opioid use and substance use disorders [7–9]. Medicaid status has also been associated with a greater likelihood of non-medical prescription opioid use and opioid-related mortality [9–11]. In a cohort of opioid-naïve commercially insured patients including 1.15% in a Medicaid managed care program, Shah et al found that initial prescriptions with days supply over 10 days or cumulative dosage over 700 morphine milligram equivalents (MME) were associated with increased likelihood of continued opioid use at 1 and 3 years after index prescription [12]. Likewise, prescription of a long acting (LA) opioid and daily dosage exceeding 90 MME was associated with persistent opioid use in a cohort study of Canadian patients receiving opioids after dental procedures [13].

ML methods may predict opioid-related endpoints by identifying complex or non-linear relationships in large datasets [14–18]. At this time, use of these techniques to identify risk factors for chronic opioid use in Medicaid patients have been limited to single states [19–21]. Other single-state retrospective cohort studies have identified factors associated with conversion from acute to chronic opioid use including back pain, previous benzodiazepine prescription, and substance abuse disorders [20]. We hypothesized that ML models trained on multi-state data could accurately identify opioid naïve patients at high risk for progression to COU and that prescription features would be key drivers of the risk scores. We developed, validated, and compared five ML models to predict progression from acute to COU amongst adult Medicaid recipients from six anonymized states. The objective of this work is to identify modifiable features associated with conversion from acute to chronic opioid use. This work may provide evidence that can guide stakeholders in their strategic policy developments.

## Methods

### Data source

Deidentified Medicaid claims were made available by the Digital Health Cooperative Research Center from 3 southeastern states, 2 western states, and 1 midwestern state from 2015–2019. Of these states, two reported data for the entire Medicaid population, one reported only fee-for service claims, and three states reported claims for Medicaid Managed Care plan enrollees, which represented 15–53% of the state’s Medicaid population. (Table A in S1 Text). Our study followed the Minimal Information for Medical AI Reporting (MINIMAR) guideline for prediction model development and validation [22]. This project utilized a de-identified limited dataset.

### Study population

**Fig 1.**
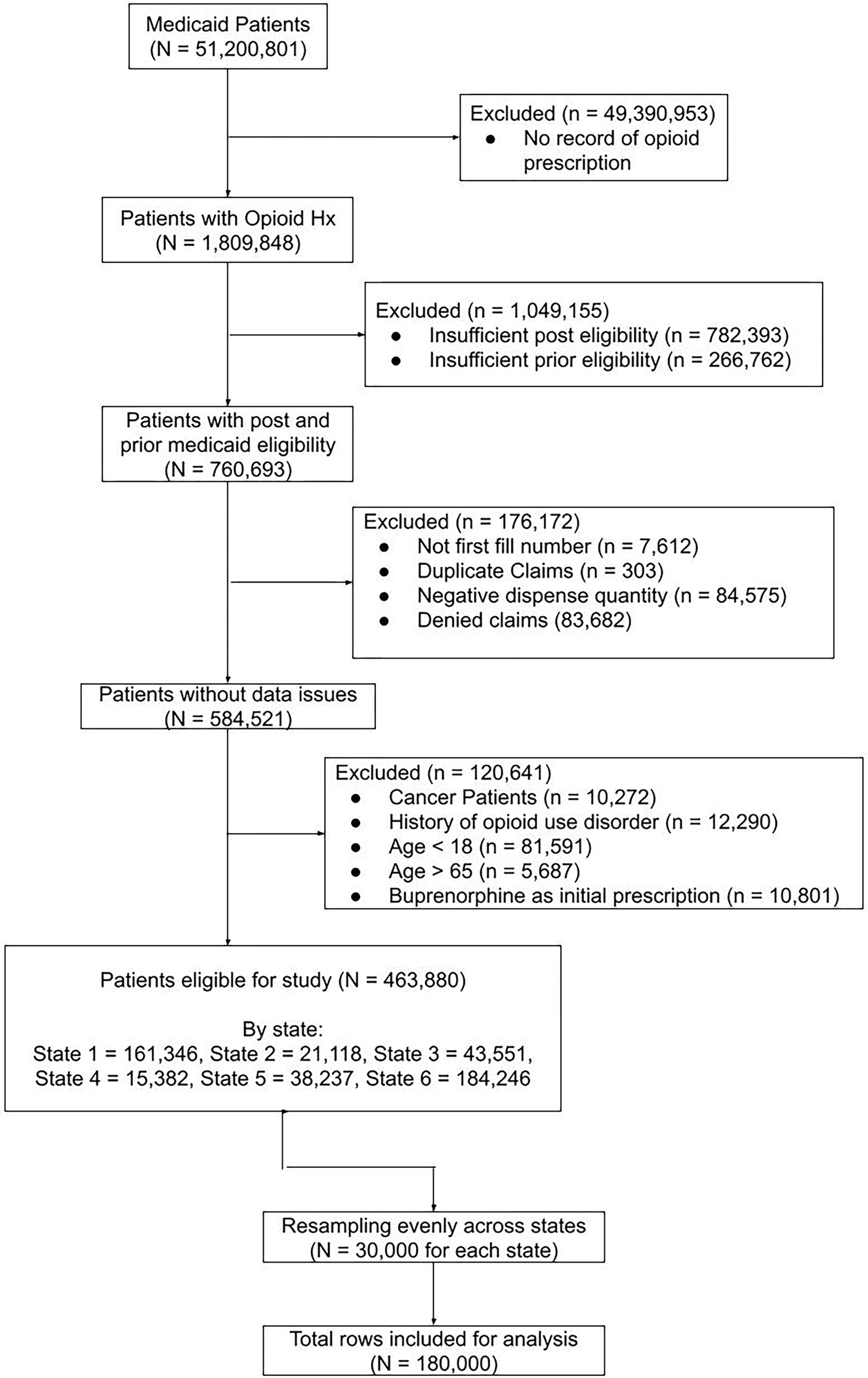
CONSORT diagram for cohort development.

Fig 1 illustrates cohort development in a CONSORT diagram. Our dataset included information from enrollment, inpatient, outpatient, dental and pharmacy Medicaid claims files. Patients aged 18 to 65 years with record of opioid prescription, 2 months of continuous enrollment prior to opioid prescription, 9 months of continuous enrollment following prescription, and opioid naïve status (defined as having 2 months without opioid prescription prior to the encounter of interest) were included. Exclusion criteria were incomplete or denied prescription claims, malignancy, history of opioid use disorder, and buprenorphine as an initial prescription given primary usage for addiction treatment. Out of 463,880 eligible patients, 30,000 were randomly sampled from each state to avoid overfitting the model on states with higher populations, effectively upsampling in states that had less than 30,000 patients. The final cohort consisted of 180,000 patients.

### Features

Demographic data collected included age and sex. Race and ethnicity data were not included due to inconsistent reporting across states. Opioid prescriptions were categorized from a list of National Drug Codes (NDCs) provided by the Center for Disease Control (Table B in S1 Text). International Disease Classification (ICD-9 and ICD-10) codes for comorbidities drawn from the Elixhauser and Charlson comorbidity indices were used in addition to those for chronic opioid abuse (Table C in S1 Text) [23,24]. Patient ZIP codes were used to generate county-level data regarding income, unemployment, education, and urban-rural classification made available by the Economic Research Service of the United States Department of Agriculture. Other features were selected with input from clinical experts regarding potential factors that may affect outcomes.

Features were removed if they were not compatible between states, had high levels of missingness, or had low levels of variance (Table D in S1 Text). For all variables included, missingness was less than 5%. We used a one-year time window prior to initial opioid prescription to look for features in the datasets. Like the other time windows, this was chosen as a tradeoff between collecting sufficient data and standardizing between different individual’s lengths of eligibility records.

Indicator features were prior prescriptions of the common anti-neuropathic pain medications gabapentin and pregabalin, in addition to the comorbidities. Prescription-related features collected included daily MME, opioid type, prescription days supply, and visit type (elective, urgent, emergent, trauma).

### Outcomes

The primary outcome of progression to COU was defined as receiving an additional opioid prescription 3 to 9 months after filling the initial prescription. Three months was chosen as the lower limit for the timeframe based on Center for Disease Control guidelines for opioid prescription, in which chronic pain was defined as pain lasting over 3 months [25]. In prior work, the upper limit for the timeframe for chronic opioid use ranged from 6 months to 1 year; 9 months was chosen as an intermediary value [26,27].

### Machine learning models

The study population was randomly split into a training cohort (80%), in which the COU prediction models were derived, and a validation cohort (20%) in which the models were applied and tested. To predict progression from opioid naïve status to COU, we developed five predictive models. A dataset consisting of 80% of the available data was used for model training. Models representing a range of computational complexity with prior success in biomedical applications were selected for inclusion: principal component analysis (PCA), logistic regression, XGBOOST, and multilayer perceptron. Hyperparameters for all models were tuned using a grid search with 5-fold cross validation, optimizing for area under the ROC curve (auROC). Missing values were replaced using mean imputation from training results. Numeric features were standardized to a zero mean and unit variance. Code has been made publicly available on GitHub (https://scikit-learn.org/stable/modules/generated/sklearn.impute.SimpleImputer.html).

### Model evaluation

One state was held out from all training data as the validation set and 20% of the remaining 5 states were held out as a test set. Model performance was evaluated by comparing mean 10-fold time-dependent incremental area under curve (iAUC) on the 20% test set. The percent of COU and non-CU in the training, testing, and validating set were similar to that in the total data set: Train: 0.292 COU, Test: 0.292 COU, Val: 0.317 COU. Precision-recall and receiver-operating characteristic curves were generated for each model using training and validation test sets.

### Variable importance

Variable importance was determined by the selection frequency for the XGBoost model, which is described elsewhere [28].

### Statistical analysis

Patient demographics were compared with unpaired t-tests for parametric data and analysis of variance (ANOVA) for parametric data, whereas the chi-square/Fisher’s exact tests were used for categorical variables. The Wilcoxon rank sum test for dependent samples was used to calculate the significance between the iAUCs. Analysis was performed using R software 4.0.3 (R Project for Statistical Computing), using two-sided tests and a significant threshold of p-value < 0.05. The parameters details can be found in the source code. All analyses were conducted using the scikit-learn machine learning module in Python and occurred between December 2018, and April 2021. Calibration curves were generated for each model in Figure A in S1 Text.

## Results

### Study population characteristics

In the cohort of 180,000 previously opioid naïve patients who were prescribed an opioid, 53,820 (29.9%) developed COU. Demographically, 113,960 (63.3%) were female with a mean age of 39.2 + 13.4 years (Table 1). COU patients were more likely to have a history of depression (16.7% vs. 11.6%, p<0.0001), chronic pulmonary disease (14.2% vs. 10.5%, p<0.0001), psychosis (8.8% vs. 7.3%, p<0.0001), diabetes (16.9% vs. 10.6%, p<0.0001), hypertension (19.0%, 10.8%, p<0.0001), and obesity (10.6% vs. 9.0% p<0.0001) compared to non-COU patients. Regarding utilization, COU patients had a greater number of mean prior Medicaid claims (46.7 vs. 42.8, p<0.0001), total prescriptions (7.5 vs 6.7, p<0.0001), days supply (26.6 vs 24.7, p<0.0001), and pills per prescription (62.4 vs. 55.6, p<0.0001) compared to non-COU patients. Prior gabapentinoids were prescribed more frequently in COU patients for pregabalin (7.0% vs. 3.4%, p<0.0001) and gabapentin (25.5% vs 15.2%, p<0.0001) compared to non-COU patients.

Characteristics of the initial opioid prescription identified for each patient are described in Table 2. Compared to patients with prescriptions limited to the acute pain period, COU patients received greater MME (33.2 vs. 23.2, p<0.0001), tablets per prescription (45.6 vs. 36.54, p<0.0001) and LA medications (2.55% vs 0.47%, p<0.0001). This difference persisted when the analysis was limited to patients with a chronic pain diagnosis (ICD-10 G89), with COU averaging 39.0 tablets per prescription and non-COU patients averaging 65.9 tablets per prescription (p<0.0001) Medications prescribed more frequently in COU patients included hydromorphone (0.56% vs. 0.42%, p = 0.0003), methadone (0.34% vs. 0.07%, p<0.0001), LA morphine (1.38% vs. 0.21%, p<0.0001), LA oxycodone (0.24% vs. 0.04%, p<0.0001), and tramadol (16.06% vs. 13.44%, p<0.0001) compared to non-COU patients. These patient demographic and prescription-level variables were incorporated as features used to train the machine learning models.

**Fig 2.**
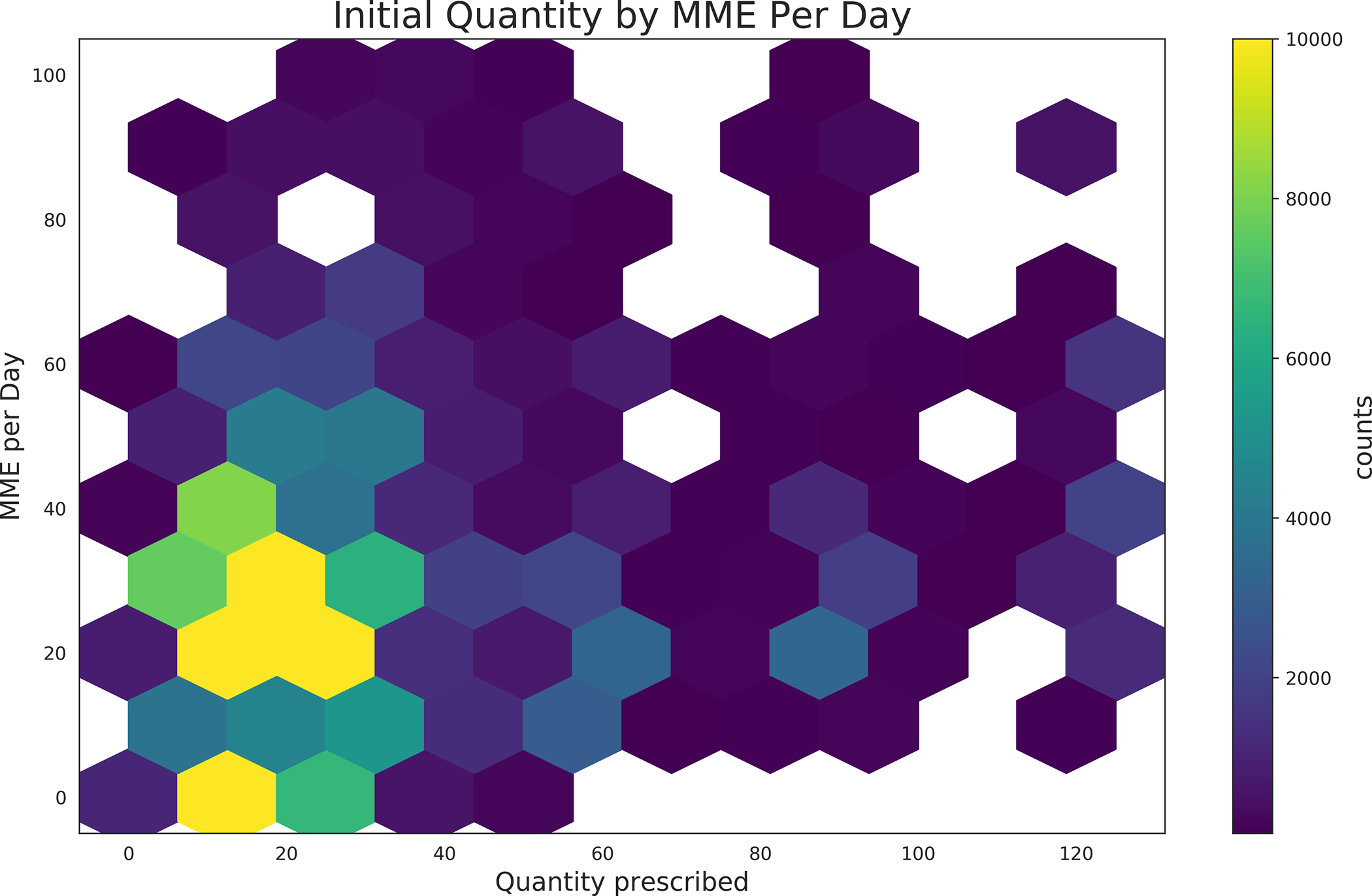

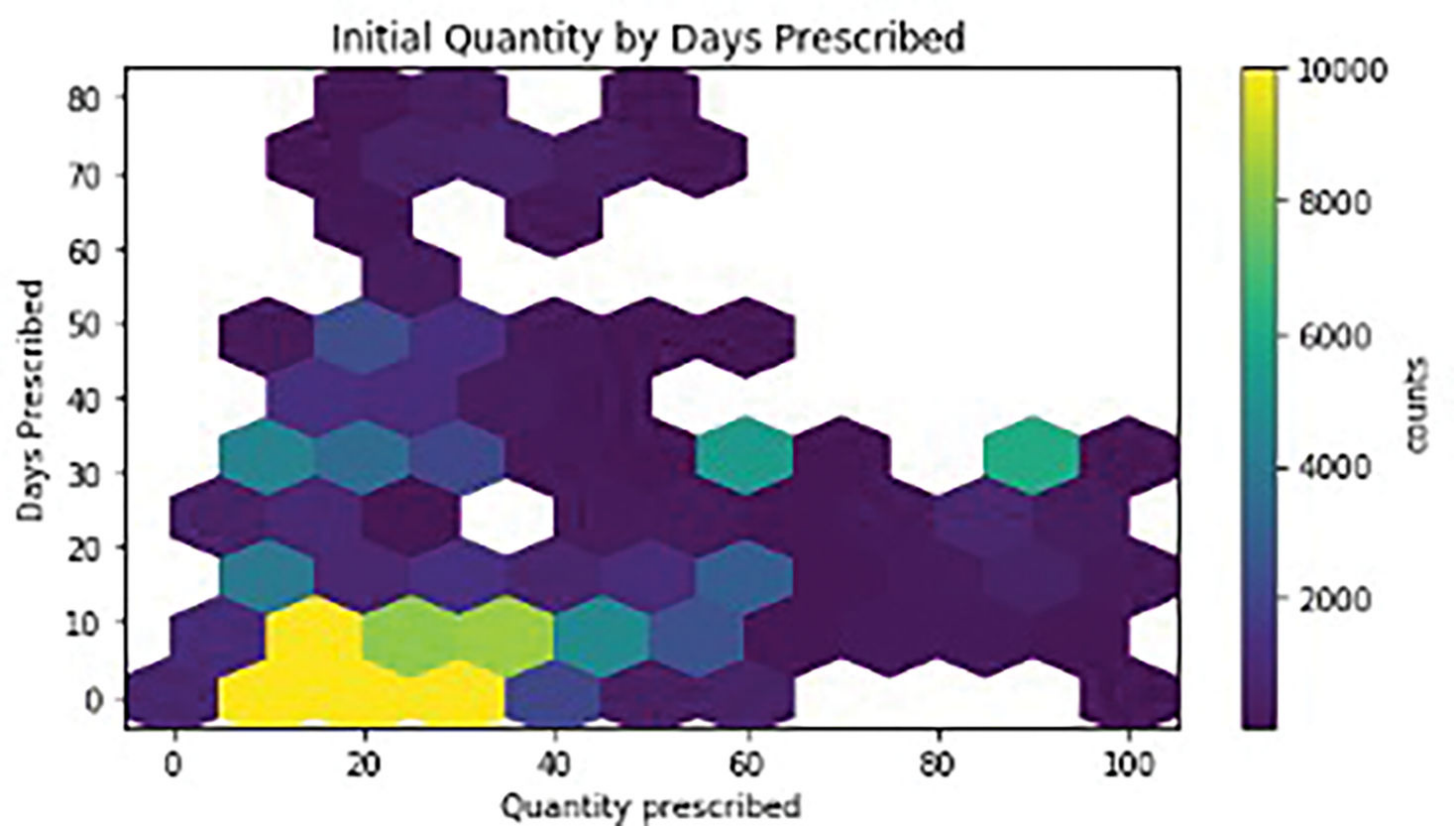
Hexagon plots illustrating the relative density of prescription quantities, days supply, and daily MME.

Hexagonal bin plots illustrating trends in prescribing practices are illustrated in Fig 2. The majority of prescriptions contained less than 40 tablets for a 10 day supply, with less up than 40 MME/day.

### Model variable importance

**Fig 3.**
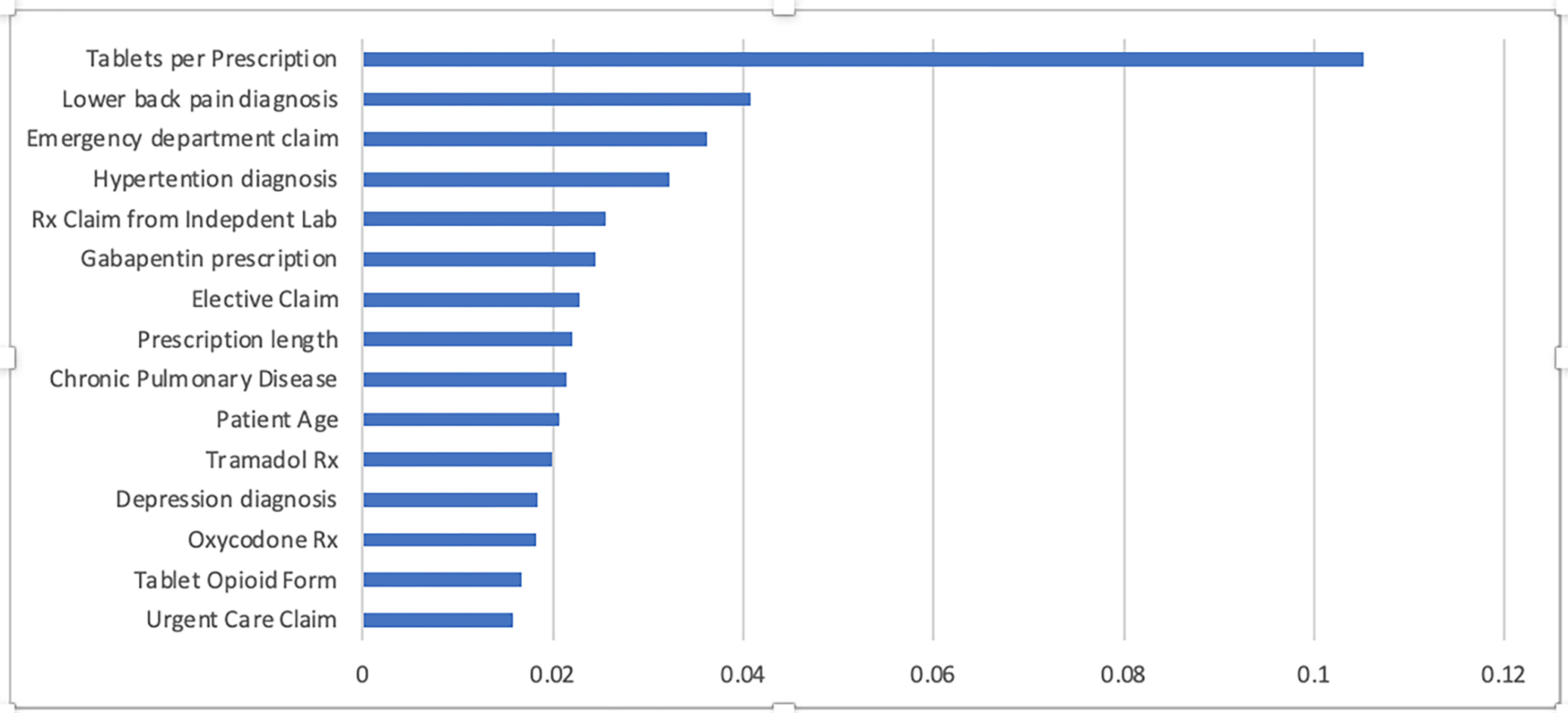
Top model features for the XGBOOST Model in descending order of importance.

The top 15 features in terms of variable importance for the XGBoost model are shown in Fig 3. Top predictors included characteristics of the initial opioid prescription including tablets per prescription, gabapentin prescription, and prescription length. The total list of variables can be found in Table E in S1 Text.

### Model performance

**Fig 4.**
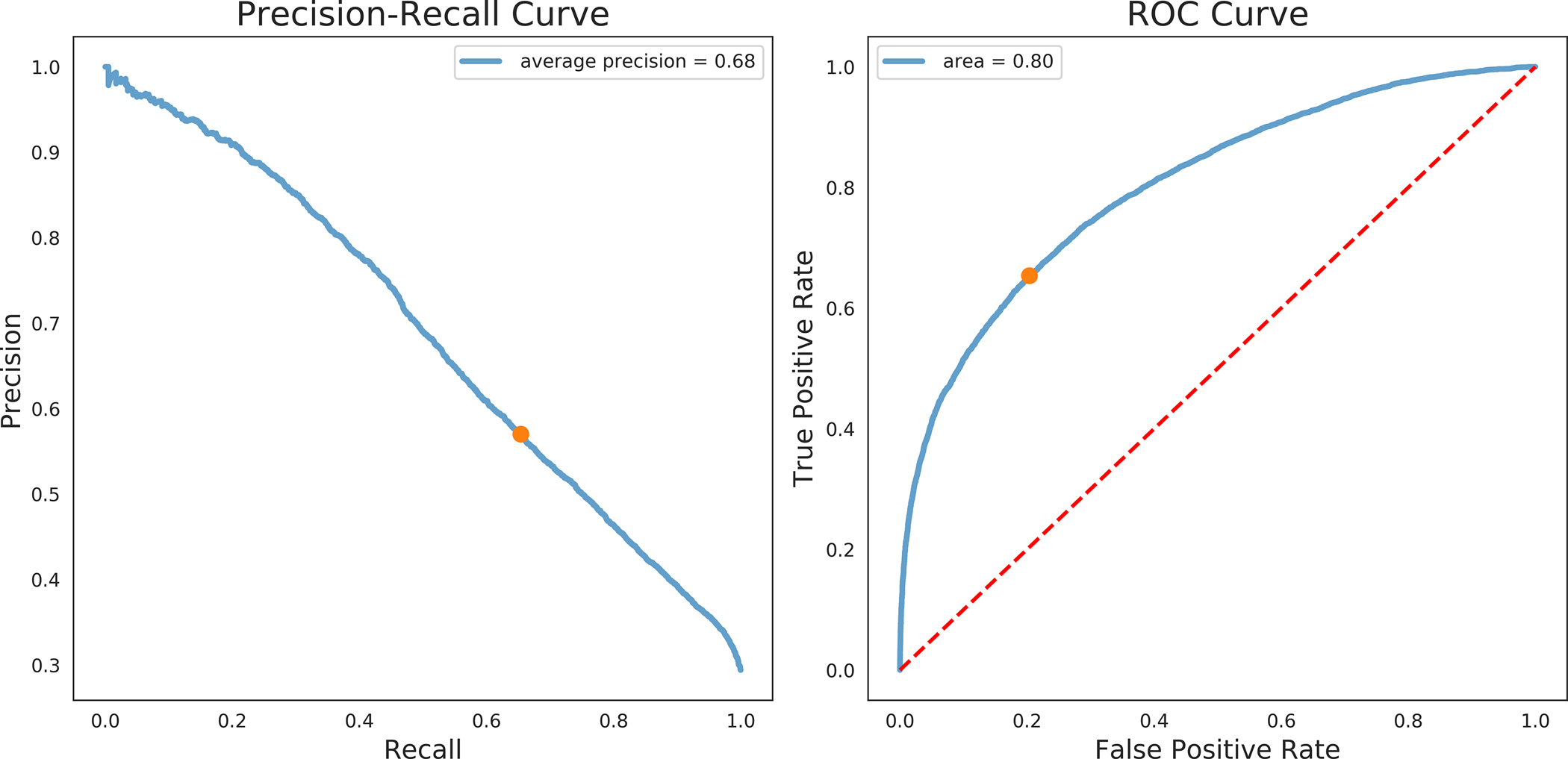

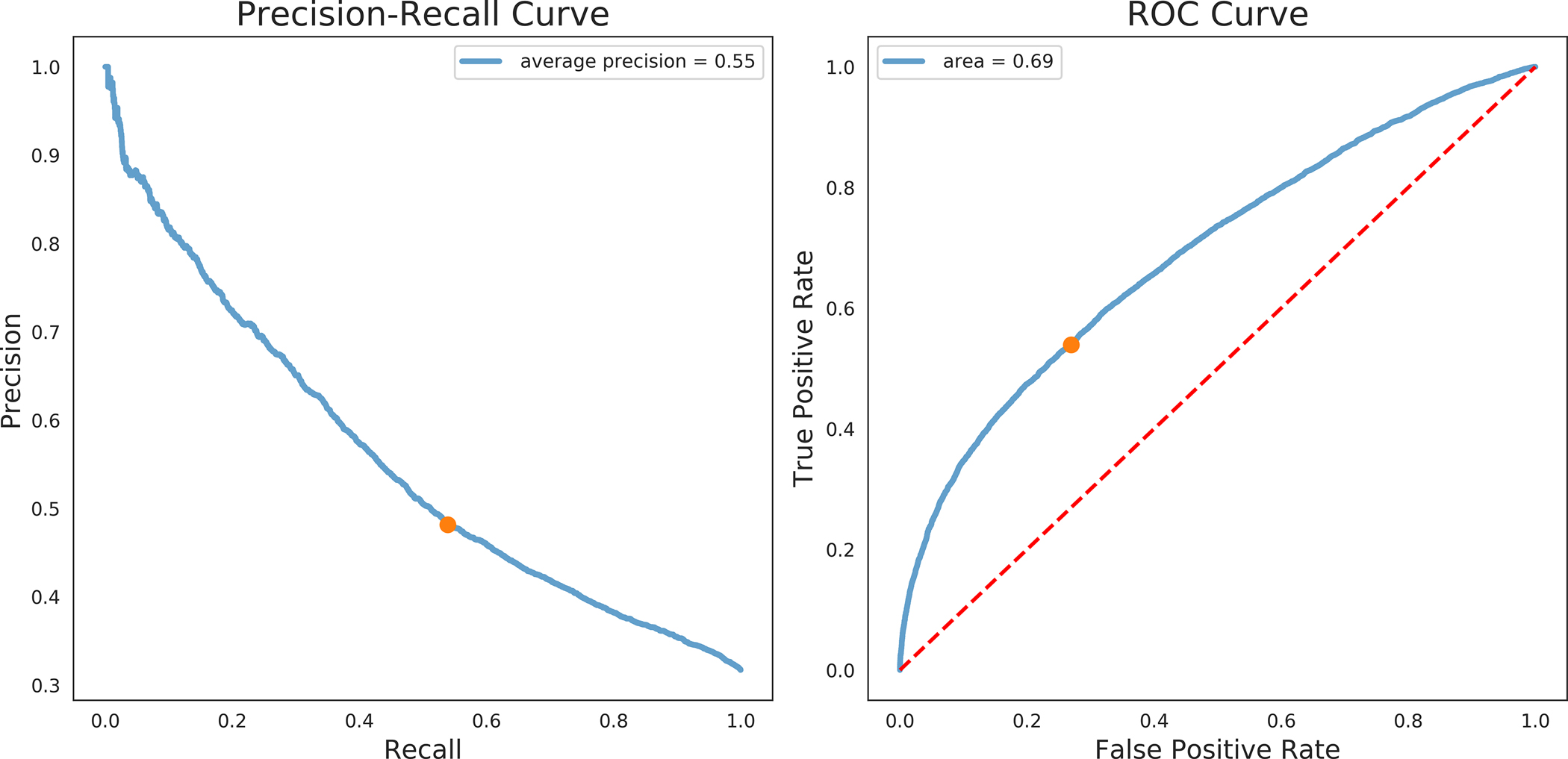
Precision-Recall and ROC Curve for the XGBoost Model for the test dataset (A) and the validation dataset (B).

**Fig 5.**
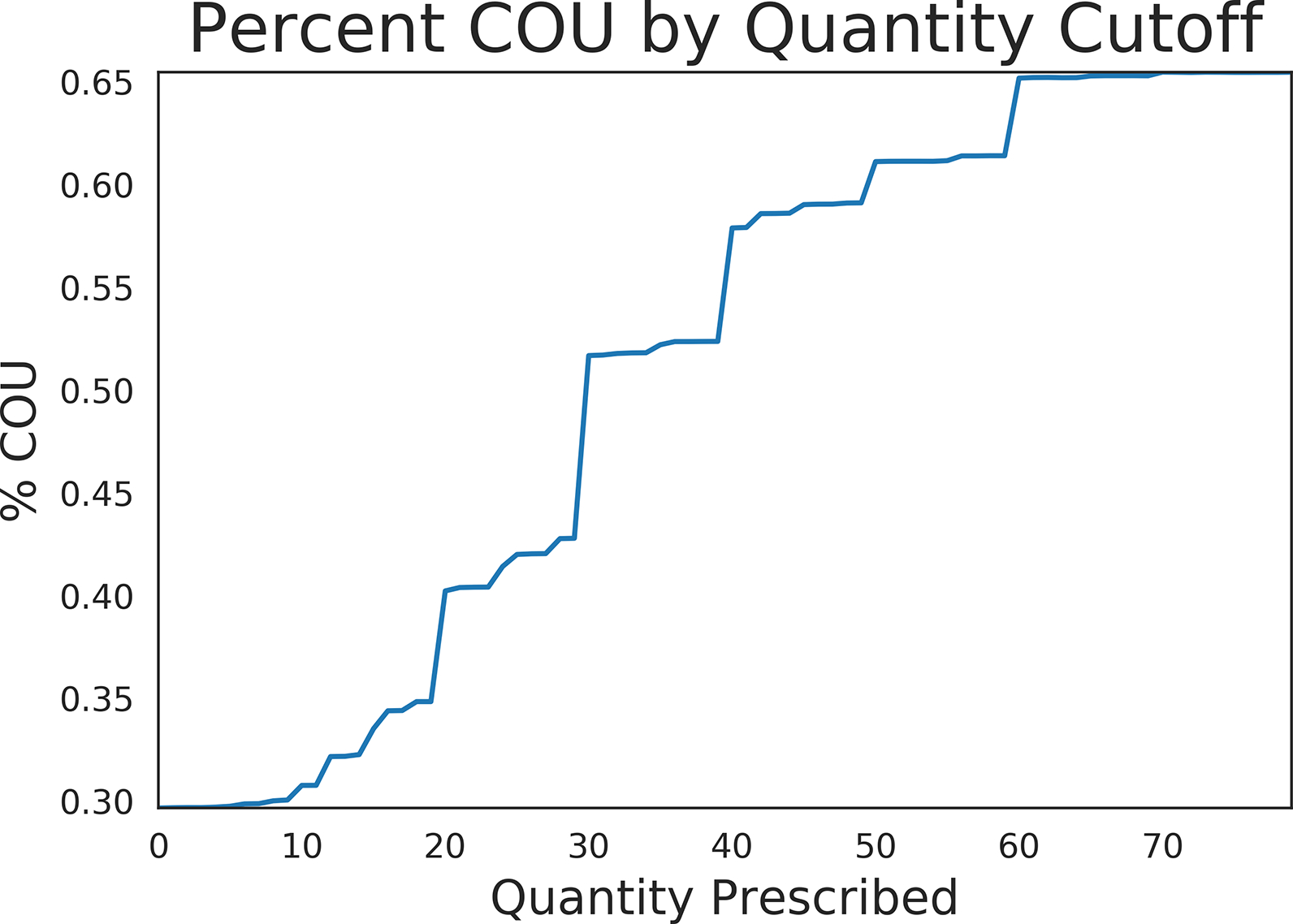
Relationship between tablet quantity and percentage of patients with incident COU.

Of the generated models, XGBOOST had the highest auROC, area under the precision recall curve (auPRC), and F1 score of 0.80, 0.68, and 0.61 respectively (Table 3). Precision recall curves and receiver operating characteristic curves for the XGBOOST model are displayed in Fig 4, with mean precision of 0.55 and auROC of 0.69 when using validation data. Fig 5 illustrates the relationship between tablet quantity and percentage of COU; notably, over 50% of COU patients in the cohort received at least 40 tablets in their initial opioid prescription.

## Discussion

In this cohort study, machine learning models based on Medicaid claims data accurately predicted progression from acute to COU among opioid naïve patients. Since Medicaid patients experience disproportionate rates of pain-related comorbidities, we hypothesized that the incidence of COU in the study cohort would be higher. Indeed, our cohort had a 29.9% rate of progression from acute to chronic opioid use, which is greater than published estimates of 10% in Medicare Part D beneficiaries [29]. COU incidence was even modestly higher than rates reported in disabled Medicare and injured Veterans Affairs populations, which ranged from 21.4% to 27% [30,31]. This may be partially explained by medical comorbidities, as patient features such as increased age, diabetes, depression, and obesity were associated with increased COU risk [32]. Social determinants of health were also relevant, as patients residing in counties with increased poverty and unemployment risk had a significantly higher risk of COU. These findings suggest that some of the risk factors previously identified in non-Medicaid populations generalized to the study cohort, although the overall incidence of COU was higher.

Within the cohort, COU risk varied by underlying chronic conditions, such as depression, diabetes, and obesity. On average, COU patients received more prescriptions, which contained a greater number of pills and MMEs for a longer length and days supply compared to non-COU patients. The ability to identify high-risk prescription features is important for policymakers and payers who currently target strategies for addressing the contribution of prescription opioids to the opioid epidemic.

In the best performing predictive model, relevant prescription-related features were tablets per prescription, previous gabapentin prescription, prescription length, and incident tramadol or LA oxycodone prescription in descending order of importance. XGBOOST, the best-performing model, is known for efficiency in large datasets and the ability to handle mixtures of numerical and categorical features. These traits may have been particularly advantageous for our cohort, which included states with diverse approaches to prescription limits: 1 state restricted tablet quantity, 4 states restricted days supply, and 1 state restricted days supply and MME.

Overall, tablet count was the most important feature in predicting COU. We demonstrate a dose-dependent relationship between tablets prescribed and COU risk; 40% of COU patients in our cohort received 20 or greater tablets in their initial prescription, while 60% of COU patients received at 50 or more tablets. This is consistent with prior studies of commercially insured opioid-naïve patients, in which increased tablet count was associated with increased risk of developing opioid use disorder [33]. A possible confounder is pain severity, as patients who present with more intense or long-lasting pain symptoms are likely to receive more initial tablets and request refills. However, our study showed that even among patients with a chronic pain diagnosis, patients progressing to COU had a higher tablet count compared to patients with chronic pain not progression to COU. Prior literature demonstrates that many patients receive excess narcotics [34], our findings suggest that reducing the quantity of opioid tablets prescribed to opioid-naïve patients may assist in reducing COU.

By using a discrete numerical cutoff, tablet quantity restrictions also have the benefit of being more easily integrated into clinical workflows. Healthcare systems could protocolize a tablet restriction or enable default tablet values within clinical decision support tools embedded in the electronic medical record, which have previously been shown to improve adherence to opioid-reduction strategies at the prescribing point of care [35–37].

Beyond individual healthcare institutions, our findings support limits on tablet quantity in opioid-related policymaking. Out of 28 states with prescribing limits for acute pain, only Colorado and Rhode Island explicitly restrict tablet quantities administered to 4 tablets per day and 20 doses overall, respectively [37,38]. The institution of tablet restrictions in Colorado was associated with a 24% decrease in mean total daily dose of opioids in patients who previously exceeded the limit [39]. Prescription length, the eighth most important feature in the model, is the most popular target for state-level opioid legislation within the cohort states and overall nationally. 24 states have enacted prescription length limits ranging from 3 to 30 days [38]. The majority of prescriptions in our cohort dispensed less than 20 day supply and were thus within compliance of at least five state limits on prescription length. Rising tablet counts may also represent compensatory behavior in prescribers faced with other dose-related restrictions; in disabled Medicare beneficiaries, Morden et al demonstrated that opioid tablet counts increased after 2011 while MME plateaued [30]. Daily MMEs are restricted in at least 3 states, but were not a relevant feature in the model.

Relevant medication-specific features including history of gabapentin prescription and incident tramadol prescription should also guide prescriber choice. Gabapentinoids have been increasingly promoted in opioid-sparing strategies in treatment of acute pain [40]. In the absence of further information regarding the clinical encounter that motivated prescription in this cohort, this finding raises questions about how gabapentinoids impact the pain trajectory of COU patients. As gabapentinoids have been associated with increased adverse events including sedation and respiratory depression when used in conjunction with opioids, further investigation is needed to evaluate the effects of co-prescribing opioids and gabapentinoids.

The higher prevalence of Tramadol prescriptions in patients progressing from acute opioid use to COU in our study merits further investigation. While Tramadol has been promoted as a safe opioid with a relatively low risk of addiction, it is notable that a weak opioid antagonist was prescribed more often in COU patients, while measures of opioid strength such as milligram morphine equivalents were not influential in the top-performing machine learning model. Taken in conjunction, our findings are aligned with recent reports that Tramadol may be a high-risk opioid for future abuse [41]. Similarly, Mudumbai et al demonstrated that postoperative time to opioid cessation was longer in veterans taking Tramadol preoperatively compared to those taking short-acting opioids [42]. These results contribute to recent literature suggesting that preferential prescribing of weak opioid agonists such as Tramadol is not equivalent to safer practice [43]. In some cases, tramadol prescription may serve as a ‘trojan horse,’ where its status as a relatively weak opioid receptor agonist may mask underlying risks regarding prolonged opioid use.

This study has important limitations. Our data represents a heterogenous collection of payer information from six states, which may not reflect the Medicaid population. However, the states include represent diverse geographical areas across the United States. Due to state policies, individual states cannot be named, which limits the interpretation of these results. However, given the size of the dataset, we generate important evidence that can guide policy decisions. Lastly, while comorbid features were selected from validated risk stratification tools, they are by no means exhaustive. Further evaluation of relevant features should be undertaken to improve the explanatory power and robustness of the model; features not easily captured in claims data such as patient access to non-opioid pain therapy may influence opioid prescribing habits. Without further data surrounding the clinical context at time of prescription, it is not possible to conclude whether an individual prescribing event was inappropriate or excessive. However, clear trends are identified through these analyses that provide insight to the prescription opioid problem amount Medicaid recipients. Our work demonstrates that machine learning models can be applied to opioid-related endpoints with good discrimination and performance; future work exploring the relationship between acute to chronic opioid use in subpopulations such as patients with acute pain conditions may further inform opioid-related policy.

## Conclusion

In a machine learning model geared toward predicting progression from acute to chronic opioid use in an opioid-naive Medicaid cohort, prescriptions with increased tablet quantity and prescription length were predictive. Despite state-level legislation, there continues to be wide variation in prescriber practices, although the majority of analyzed prescriptions dispensed fewer than 40 tablets for a 20 day supply. Future research should evaluate the effects of restricting opioid tablet quantity on COU incidence.

## Supplementary Material

Supplement

## Figures and Tables

**Table 1.** Patient demographics stratified by acute and chronic opioid users.

Variables	Total		Acute		Chronic		p-value
	N	%	N	%	N	%	
**Number of Patients**	180,000	100	126,180	70.1	53820	29.9	
**Age, Mean (SD)**	39.18	13.36	37.33	13.22	43.52	12.67	P<0.0001
**Sex**							
Female	113960	63.3	79974	63.4	33986	63.2	0.3918
Male	66036	36.7	46202	36.6	19834	36.9	0.3860
**Comorbidities**							
Depression	23647	13.1	14644	11.6	9002	16.7	P<0.0001
Chronic pulmonary disease	20892	11.6	13203	10.5	7689	14.2	P<0.0001
Psychoses	13907	7.7	9177	7.3	4730	8.8	P<0.0001
Diabetes*	22504	12.5	13388	10.6	9116	16.9	P<0.0001
Hypertension*	23861	13.3	13605	10.8	10256	19.0	P<0.0001
Obesity	17097	9.5	11375	9.0	5722	10.6	P<0.0001
Substance use disorder	6784	3.8	4601	3.6	2183	4.1	P<0.0001
**Prior Medicaid claims, mean (SD)**	43.97	62.72	42.81	61.65	46.67	65.06	P<0.0001
**Prior Prescription Features**							P<0.0001
Total prescriptions, mean (SD)	7.99	7.01	7.72	6.77	8.64	7.52	P<0.0001
Days per prescription, mean (SD)	25.27	11.01	24.69	11.20	26.63	10.44	P<0.0001
Total days for prescriptions	552.32	756.18	535.43	745.75	591.87	778.63	P<0.0001
Pills per prescription	57.60	549.65	55.56	470.02	62.39	701.57	0.0282
Patient County Information**							
Median Household Income	56078	11750	56093	11619	56043	12052	0.4562
Percent in Poverty	14.42	4.53	14.39	4.47	14.50	4.66	P<0.0001
Unemployment rate	4.24	1.05	4.21	1.03	4.30	1.08	P<0.0001
County urban categorization^&^							
Rural-urban continuum code	2.39	1.94	2.34	1.90	2.48	2.01	P<0.0001
Urban influence code	2.39	2.33	2.34	2.27	2.51	2.47	P<0.0001
**Previous Multimodal Prescriptions**							
Pregabalin	8135	4.51	4340	3.42	3795	7.00	P<0.0001
Gabapentin	32871	18.30	19093	15.22	13778	25.53	P<0.0001

* Includes complicated and uncomplicated diagnoses

& National Center for Health Statistics Urban-Rural Classification scheme from 1 to 6, with 1 being remote rural and 6 being inner-city, 2013

** 2018

**Table 2.** Characteristics of the initial opioid prescription for each patient.

Variables	Total		Acute		Chronic		p-value
	N	%	N	%	N	%	
**Total Prescriptions**	180,000	100	126,180	70.1	53820	29.9	
**Daily MME, Mean (SD)**	29.40	26.68	27.87	23.17	32.98	33.21	P<0.001
**Institutional Information**							
**Elective claim**	15974	8.88	11583	9.19	4391	8.13	P<0.001
**Emergency Claim**	23507	13.06	16662	13.22	6845	12.68	0.004
**Trauma Center Claim**	425	0.24	316	0.25	109	0.20	0.074
**Urgent claim**	4139	2.30	3028	2.40	1111	2.06	P<0.001
**Opioid Type**							
Codeine	14156	7.8	10790	8.56	3366	6.23	P<0.001
Fentanyl LA	396	0.22	171	0.14	224	0.42	P<0.001
Hydrocodone SA	88565	49.20	62994	50.00	25571	47.35	P<0.001
Hydromorphone SA	826	0.46	525	0.42	301	0.56	P<0.001
Methadone	271	0.15	89	0.07	182	0.34	P<0.001
Morphine LA	1006	0.56	258	0.21	748	1.38	P<0.001
Morphine SA	363	0.20	227	0.18	136	0.25	0.005
Oxycodone LA	178	0.10	46	0.04	132	0.24	P<0.001
Oxycodone SA	48336	26.85	33858	26.87	14478	26.81	0.810
Pentazocine	44	0.02	18	0.01	25	0.05	P<0.001
Tramadol SA	25611	14.23	16940	13.44	8672	16.06	P<0.001
**Prescription Information**							
Prescription length (days), N (SD)	12.94	14.67	11.57	14.65	16.15	14.20	P<0.001
Tablets per Prescription, N (SD)	32.81	40.53	26.77	36.54	46.94	45.59	P<0.001
Long Acting	1969	1.09	592	0.47	1377	2.55	P<0.001
Short Acting	178021	98.90	125401	99.52	52620	97.44	P<0.001

* SA: Short Acting

* LA: Long Acting

**Table 3.** Model performance based on receiver operating characteristics, accuracy, precision, and recall. The XGBOOST model’s performance on validation data is included.

Model Type	AuROC	auPRC	F1 Score	Accuracy	Precision	Recall
PCA Logistic Regression *	0.63	0.48	0.44	0.59	0.37	0.56
**Logistic Regression**	0.72	0.54	0.53	0.69	0.47	0.60
**ElasticNet**	0.71	0.53	0.52	0.68	0.47	0.59
**Multi-layer Perceptron**	0.73	0.59	0.52	0.68	0.47	0.59
**XGBOOST**	0.80	0.68	0.61	0.75	0.57	0.65
**XGBOOST (validation)**	0.69	0.55	0.51	0.67	0.48	0.54

## Data Availability

The raw data used in this study contain personal health information and therefore cannot be released to the public, readers may request access from Nilpa Shah (nilpa.shah@stanford.edu). However, all the code used to generate the analysis are available via GitHub (https://github.com/drgojo/mcaid-opioid).
