# Supplementary material for: Prescription quantity and duration predict progression from acute to chronic opioid use in opioid-naïve Medicaid patients
Source: PLOS Digit Health. Author manuscript; Available in PMC 2022 Oct 5. (PMC9534483; doi:10.1371/journal.pdig.0000075)
Supplement: Supplementary file 1 [file NIHMS1836991-supplement-Supplement.docx]

**Supplementary Material**

eTable 1. State Demographics

eTable 2. Opioids selected for inclusion, based on the National Drug Codes provided by the Center for Disease Control.

eTable 3. ICD-9 and ICD-10 codes used for demographic data collection.

eTable 4. Full List of Features by Code and Description

**S1 Table. State Demographics**

| STATE | AGE | Metropolitan Area (%) | Bachelors or higher | Median Household Income (County) | Unemployment rate(%) | Female (%) | Urban influence | High School Degree only (%) |
| --- | --- | --- | --- | --- | --- | --- | --- | --- |
| 1 | 44.0 | 72.2% | 25.8 | $ 56,668.11 | 4.32 | 62.9% | 77.6% | 30.9 |
| 2 | 38.5 | 82.1% | 29.5 | $ 59,273.13 | 4.02 | 61.9% | 84.6% | 28.7 |
| 3 | 39.1 | 76.5% | 25.7 | $ 54,400.70 | 4.49 | 65.7% | 78.9% | 32.1 |
| 4 | 35.7 | 87.4% | 28.0 | $ 55,504.77 | 3.85 | 67.5% | 90.7% | 29.8 |
| 5 | 39.9 | 75.3% | 25.8 | $ 54,321.99 | 4.42 | 59.3% | 77.2% | 31.7 |
| 6 | 37.0 | 81.7% | 27.5 | $ 56,479.31 | 4.23 | 64.6% | 86.4% | 30.7 |

**S2 Table. Opioids selected for inclusion, based on the National Drug Codes provided by the Center for Disease Control.**

| Opioid |
| --- |
| Buprenorphine |
| Butorphanol |
| Codeine |
| Dihydrocodeine |
| Fentanyl LA |
| Fentanyl SA |
| Hydrocodone LA |
| Hydrocodone SA |
| Hydromorphone LA |
| Hydromorphone SA |
| Levomethadyl |
| Levorphanol |
| Meperidine |
| Methadone |
| Morphine LA |
| Morphine SA |
| Opium |
| Oxycodone LA |
| Oxycodone SA |
| Oxymorphone LA |
| Oxymorphone SA |
| Pentazocine |
| Propoxyphene |
| Tapentadol LA |
| Tapentadol SA |
| Tramadol LA |
| Tramadol SA |

**S3 Table. ICD-9 and ICD-10 codes used for demographic data collection.**

|  | ICD-9 | ICD-10 |
| --- | --- | --- |
| Congestive Heart Failure | 39891, 40211, 40291, 40411, 40413, 40491, 40493, 428 | I099,  I110,  I130,  I132, I255, I420, I425, I426, I427, I428, I429, I43, I50, P290 |
| Cardiac Arrhythmia | 41610, 42611, 42613, 4262, 4263, 4264, 4265, 4266, 4267, 4268, 4270, 4272, 42731, 42760, 4279, 7850, V450, V533 | I441, I442, I443, I456, I459, I47, I48, I49, R001, R008, T821, Z450, Z950 |
| Valvular Disease | 0932, 394, 395, 396, 3971, 3979, 424, 7463, 7464, 7465, 7466, V422, V433 | A520, I05, I06, I07, I08, I091, I098, I34, I35, I36, I37, I38, I39, Q230, Q231, Q232, Q233, Z952, Z953, Z954 |
| Pulmonary Circulation Disorder | 416, 4179 | I26, I27, I280, I288, I289 |
| Peripheral Vascular Disorder | 440, 4412, 4414, 4417, 4419, 4431, 4432, 4433, 4434, 4435, 4436, 4437, 4438, 4439, 4471, 5571, 5579, V43 | I70, I71, I731, I738, I739, I771, I790, I792, K551, K558, K559, Z958, Z959 |
| Hypertension | 4011, 4019 | I10, I11, I12, I13, I15 |
| Paralysis | 3420, 3421, 3429, 343, 344 | G041, G114, G801, G802, G81, G82, G830, G831, G832, G833, G834, G839 |
| Other Neurological Disorder | 3319, 3320, 3334, 3335, 334, 335, 340, 3411-3419, 345, 3481, 3483, 7803, 7843 | G10-G13, G20-G22, G254, G255, G312, G318, G319, G32, G35-G37, G40, G41, G931, G934, R470, R56 |
| Chronic Pulmonary Disease | 4168, 4169, 490-494, 505, 5064, 5081, 5088 | I278, I279, J40-J47, J60-J67, J684, J701, J703 |
| Diabetes | 2500-2509 | E100-E149 |
| Hypothyroidism | 243, 2440 – 2442, 2448, 2449 | E00-E03, E890 |
| Renal Failure | 40311, 40391, 40412, 40492, 585, 586, V420, V451, V560, V568 | I120, I131, N18-19, N250, Z490-Z492, Z992 |
| Liver Disease | 07032, 07033, 07054, 456-4562, 5710, 5712-5719, 5723, 5728, V427 | B18, I85, I864, I982, K70, K711, K713-K715, K717, K72-K74, K760, K762-K769, Z944 |
| Gastric Ulcer | 53141, 53151, 531615317, 53191, 53241, 53251, 53261, 5327, 53191, 5324153251, 53261, 532753291, 53341, 53351, 53361, 5337, 53391, 53441, 53451, 534615347, 53491 | K257, K259, K267, K269, K277, K279, K287, K289 |
| Human Immunodeficiency Virus | 042 – 044 | B20-B22, B24 |
| Lymphoma | 200, 201, 2020–2023, 2025-2030, 2038, 2386, 273 | C81-C85, C88, C96, C900, C902 |
| Metastatic Cancer | 196-199 | C77-C80 |
| Solid Tumor without Metastasis | 140-195, V10 | C00-C34, C37-C41, C43, C45-C58, C60-C76, C97 |
| Rheumatoid Arthritis | 7010, 710, 714, 720, 725 | L940, L941, L043, M05, M06, M08, M120, M123, M30, M310-M313, M32-M35, M45, M461, M468, M469 |
| Coagulopathy | 286, 2871, 2873-2875 | D65-D68, D691, D693-D696 |
| Obesity | 2780 | E66 |
| Weight Loss | 260-263, 7832 | E40-E46, R634, R64 |
| Fluid and Electrolyte disorders | 276 | E222, E86, E87 |
| Anemia | 2800-2809, 281, 2859 | D500, D508, D509, D51-53 |
| Alcohol Abuse | 291, 2912, 2915-2919, 3039, 3050, V113 | F10, E52, G621, I 426, K292, K700, K703, K709, T51, Z502, Z715, Z722 |
| Drug Abuse | 2920, 29282-28289, 2929, 3040, 3052-3058, 29282-29289, 2929, 3040, 3052-3059 | F11-F16, F18, F19, Z715, Z722 |
| Psychoses | 295-298, 2991 | F20, F22-F25, F28, F29, F302, F312, F315 |
| Depression | 3004, 30112, 3090, 3091, 311 | F204, F313-F315, F32, F33, F341, F412, F432 |
| Opioid Use Disorder | 30550, 3040, 3047 | F111, F112, F119 |

**S4 Table. Variable Missingness**

| Variable | Number Missing |
| --- | --- |
| ABUSE_DETER | 10 |
| acetaminophen_ndcs | 35136 |
| AGE | 0 |
| aids_hiv | 0 |
| alcohol_abuse | 0 |
| ambulance_claim | 15368 |
| ambulatory_surgery_claim | 15368 |
| amitriptalin_ndcs | 35136 |
| blood_loss_anemia | 0 |
| buprenorphine_ndcs | 35136 |
| bupropion_ndcs | 35136 |
| capsaicin_ndcs | 35136 |
| cardiac_arrhythmia | 0 |
| chronic_pulmonary_disease | 0 |
| coagulopathy | 0 |
| congestive_heart_failure | 0 |
| dacomitinib_ndcs | 35136 |
| deficiency_anemia | 0 |
| depression | 0 |
| desipramine_ndcs | 35136 |
| diabetes_complicated | 0 |
| diabetes_uncomplicated | 0 |
| diclofenac_ndcs_x | 35136 |
| diclofenac_ndcs_y | 35136 |
| doxipen_ndcs | 35136 |
| drug_abuse | 0 |
| Drug_Codeine | 0 |
| Drug_Fentanyl LA | 0 |
| Drug_Hydrocodone SA | 0 |
| Drug_Hydromorphone SA | 0 |
| Drug_Methadone | 0 |
| Drug_Morphine LA | 0 |
| Drug_Morphine SA | 0 |
| Drug_Oxycodone LA | 0 |
| Drug_Oxycodone SA | 0 |
| Drug_Pentazocine | 0 |
| Drug_Tramadol SA | 0 |
| duloxetine_ndcs | 35136 |
| ELIGIBILITY_TIME | 0 |
| emergency_room_claim | 15368 |
| fluid_and_electrolyte_disorders | 0 |
| fluoxetine_ndcs | 35136 |
| gabapentin_ndcs | 35136 |
| gastric_ulcer | 0 |
| GENDER_F | 0 |
| GENDER_M | 0 |
| hasInstitutional | 0 |
| Hispanic | 0 |
| home_claim | 15368 |
| hypertension_complicated | 0 |
| hypertension_uncomplicated | 0 |
| hypothyroidism | 0 |
| ibuprofen_ndcs | 35136 |
| imipramine_ndcs | 35136 |
| init_diag_E11 | 0 |
| init_diag_E66 | 0 |
| init_diag_E78 | 0 |
| init_diag_F17 | 0 |
| init_diag_F32 | 0 |
| init_diag_F41 | 0 |
| init_diag_G89 | 0 |
| init_diag_I10 | 0 |
| init_diag_J45 | 0 |
| init_diag_K04 | 0 |
| init_diag_K08 | 0 |
| init_diag_K21 | 0 |
| init_diag_K80 | 0 |
| init_diag_L02 | 0 |
| init_diag_L03 | 0 |
| init_diag_M25 | 0 |
| init_diag_M54 | 0 |
| init_diag_M79 | 0 |
| init_diag_N20 | 0 |
| init_diag_N39 | 0 |
| init_diag_N83 | 0 |
| init_diag_R07 | 0 |
| init_diag_R10 | 0 |
| init_diag_R11 | 0 |
| init_diag_R51 | 0 |
| init_diag_S62 | 0 |
| init_diag_S82 | 0 |
| init_diag_Z68 | 0 |
| init_diag_Z79 | 0 |
| init_diag_Z87 | 0 |
| init_diag_Z88 | 0 |
| InitDental | 0 |
| InitHomeHealth | 0 |
| INITIAL_CLAIM_TYPE_12 | 0 |
| INITIAL_RX_AMOUNT_BILLED | 0 |
| INITIAL_RX_AMOUNT_PAID | 0 |
| INITIAL_RX_LENGTH | 0 |
| INITIAL_RX_NDC_CODE | 0 |
| INITIAL_RX_QUANTITY | 0 |
| InitIndependentClinic | 0 |
| InitIndipendantLab | 0 |
| InitInpatient | 0 |
| InitLongTermCare | 0 |
| InitMedEquip | 0 |
| InitOutpatient | 0 |
| InitPharmacy | 0 |
| InitPhysician | 0 |
| InitRehabilitation | 0 |
| InitTransportAmb | 0 |
| InitTransportNonAmb | 0 |
| inpatient_claim | 15368 |
| InstitutionalElective | 0 |
| InstitutionalEmergency | 0 |
| InstitutionalNewborn | 0 |
| InstitutionalTraumaCenter | 0 |
| InstitutionalTraumaNotAvail | 0 |
| InstitutionalUrgent | 0 |
| ketorolac_ndcs | 35136 |
| lidocaine_ndcs | 35136 |
| liver_disease | 0 |
| LongShortActing_LA | 0 |
| LongShortActing_SA | 0 |
| lymphoma | 0 |
| Master_Form_Capsule | 0 |
| Master_Form_Capsule, Extended Release | 0 |
| Master_Form_Patch, Extended Release | 0 |
| Master_Form_Solution | 0 |
| Master_Form_Tablet | 0 |
| Master_Form_Tablet, Extended Release | 0 |
| MED | 10 |
| Med_HH_Income_Percent_of_State_Total_2018 | 3668 |
| Median_Household_Income_2018 | 3668 |
| mental_health_claim | 15368 |
| metastatic_cancer | 0 |
| milnacipran_ndcs | 35136 |
| naltrexone_ndcs | 35136 |
| naproxen_ndcs | 35136 |
| nortryptaline_ndcs | 35136 |
| obesity | 0 |
| opioid_disorder | 0 |
| other_neurological_disorder | 0 |
| OUTCOME | 0 |
| outpatient_claim | 15368 |
| paralysis | 0 |
| paroxetine_ndcs | 35136 |
| PCTPOVALL_2018 | 3668 |
| Percent_bachelors_or_higher_1418 | 3591 |
| Percent_less_than_high_school_1418 | 3591 |
| Percent_only_high_school_1418 | 3591 |
| Percent_some_college_1418 | 3591 |
| peripheral vascular_disorder | 0 |
| PRE_NUM_POLYPHARMACY | 35136 |
| PRE_RX_BILLED_AVG | 35136 |
| PRE_RX_BILLED_SUM | 35136 |
| PRE_RX_DAYS_AVD | 35136 |
| PRE_RX_DAYS_SUM | 35136 |
| PRE_RX_PAID_AVG | 35136 |
| PRE_RX_PAID_SUM | 35136 |
| PRE_RX_QTY_AVG | 35136 |
| PRE_RX_QTY_SUM | 35136 |
| pregabalin_ndcs | 35136 |
| psychoses | 0 |
| pulmonary_circulation_disorder | 0 |
| quinidine_ndcs | 35136 |
| renal_failure | 0 |
| rheumatoid_arhritis | 0 |
| Rural_urban_continuum_code_2013 | 3591 |
| solid_tumor_wo_metastasis | 0 |
| STATE | 0 |
| TOTAL_PRE_CLAIMS | 15368 |
| TOTAL_RXS | 35136 |
| Unemployment_rate_2018 | 3591 |
| Urban_influence_code_2013 | 3591 |
| urgent_care_claim | 15368 |
| valvular_disease | 0 |
| venlafaxin_ndcs | 35136 |
| weight_loss | 0 |

**S5 Table. Full List of Features by Code and Description**

| Feature Category or Name | Feature Description |
| --- | --- |
| County-level Features (based on 2018 data) | Median household income in county |
|  | Median Household Income in county as a percent of the state total |
|  | Rural-urban classification of county |
|  | Urban influence |
|  | Unemployment rate of county |
|  | Percent of county in poverty |
|  | Percent of county with less than high school as highest level of education |
|  | Percent of county with only high school as highest level of education |
|  | Percent of county with some college as highest level of education |
|  | Percent of county with a bachelors or higher as highest level of education |
| Prior Pain-Related Medication Prescriptions & Prescription Characteristics | Amitriptyline |
|  | Naproxen |
|  | Lidocaine |
|  | Fluoxetine |
|  | Gabapentin |
|  | Nortriptyline |
|  | Diclofenac |
|  | Ibuprofen |
|  | Duloxetine |
|  | Paroxetine |
|  | Ketorolac |
|  | Doxipen |
|  | Bupropion |
|  | Venlafaxine |
|  | Pregabalin |
|  | Total Number of prescriptions in pre period |
|  | Number of unique drugs prescribed in pre period |
|  | Average cost of prescription in pre period |
|  | Average quantity dispensed per prescription in pre period |
|  | Average days prescribed per prescription in pre period |
|  | Average paid out per prescrption in pre period |
|  | Sum total quantity dispensed in pre period |
|  | Sum total paid out for prescriptions in pre period |
|  | Sum total billed for prescriptions in pre period |
|  | Sum total of days of prescriptions given in pre period |
| Initial Diagnosis at Time of Prescription | Diagnosis given at initial prescription event |
|  | Type 2 Diabetes Melitus |
|  | Long Term Drug Therapy |
|  | Joint Disorder |
|  | Soft Tissue Disorder |
|  | Anxiety Disorder |
|  | Abdominal and Pelvic Pain |
|  | Nicotine Dependence |
|  | Dorsalgia |
|  | Pain |
|  | Pain in throat and chest |
|  | Personal history of diseases and conditions |
|  | Disorders of lipoprotein metabolism and other lipidemias |
|  | Essential (primary) hypertension |
| Demographics | Male gender |
|  | Female gender |
|  | Age at time of initial prescription |
|  | Cumulative duration of Medicaid eligibility at time of initial prescription |
|  | Hispanic ethnicity |
| Comorbidities (ICD-9) | Uncomplicated hypertension |
|  | Complicated diabetes |
|  | Rheumatoid arthritis |
|  | Liver disease |
|  | Anemia |
|  | Coagulopathy |
|  | Complicated hypertension |
|  | Peripheral vascular disease |
|  | Alcohol abuse |
|  | Chronic obstructive pulmonary disease |
|  | Cardiac arrhythmia |
|  | Valvular disease |
|  | Renal failure |
|  | Uncomplicated diabetes |
|  | Hypothyroidism |
|  | Psychosis |
|  | Congestive heart failure |
|  | Fluid and electrolyte disorder |
|  | Paralysis |
|  | Depression |
|  | Other neurological disorder |
|  | Obesity |
|  | Weight loss |
| Index Prescription Features | Amount paid out for initial prescription |
|  | Amount billed for initial prescription |
|  | SA opioid prescribed |
|  | LA opioid prescribed |
|  | Hydrocodone SA prescribed |
|  | Oxycodone SA prescribed |
|  | Codeine prescribed |
|  | Tramadol SA prescribed |
|  | MME/day of prescription |
|  | Number of pills prescribed |
|  | Length of prescription in days |
|  | Opioid given in tablet forumulation |
| Prior Claim Types | Emergency room claim |
|  | Inpatient claim |
|  | Mental health claim |
|  | Ambulance claim |
|  | Home claim |
|  | Urgent care claim |
|  | Ambulatory surgery claim |
|  | Outpatient claim |
| Initial Claim Types | Dental |
|  | Home Health |
|  | Ambulance transport |
|  | Independent Lab |
|  | Outpatient |
|  | Pharmacy |
|  | Physician |
| Institutional Claim Features | Indicator for if an institutional claim is present in record |
|  | Urgent institutional claim |
|  | Emergency institutional claim |
|  | Elective institutional claim |

**S1 Fig. Model Calibration Curves for PCA (A), XGBoost (B), Logistic Regression (C), ElecticNet (D), and MLP (E)**

A

B

C

D

E
